# May positron emission tomography reveal ectopic or active thymus in preoperative evaluation of non-thymomatous myasthenia gravis?

**DOI:** 10.1186/s13019-014-0146-0

**Published:** 2014-09-05

**Authors:** Tommaso Claudio Mineo, Vincenzo Ambrogi, Orazio Schillaci

**Affiliations:** Thoracic Surgery Division, Multidisciplinary Myasthenia Gravis Unit, Department of Experimental Medicine and Surgery, Policlinico Tor Vergata University, Tor Vergata University Rome, Rome, Italy; Department of Biomedicine and Prevention, Policlinico Tor Vergata University, Tor Vergata University Rome, Rome, Italy

**Keywords:** Thymectomy, Myasthenia gravis, Positron emission tomography

## Abstract

**Background:**

In myasthenia gravis (MG) both native and ectopic thymic tissue containing germinal centers should show greater metabolism compared to adjacent tissues. We evaluated whether preoperative standardized uptake value (SUV) of 18fluoro-deoxy-glucose on Positron Emission Tomography (PET) might be increased and correlated with the presence of native or ectopic germinal centers.

**Methods:**

From 2005 to 2012 we performed extended thymectomy in 68 patients with non-thymomatous MG. All patients underwent PET-scan preoperatively and one-year postoperatively. SUVs were assessed in thymic and perithymic regions. Then it was matched with same-age, non-MG and non-neoplastic control group and finally correlated with presence of germinal centers in native thymus or in the ectopic tissue found in the surgical specimens.

**Results:**

Mean SUV was significantly increased in MG patients compared to control group. Thymic SUV was significantly higher in presence of thymic germinal centers [3.5 (2.4-5.0) Vs 2.1 (1.4-2.5), p = 0.021] while perithymic SUV was significantly higher in presence of ectopic germinal centers [3.1 (2.7-3.5) Vs 1.3 (0.9-1.7), p = 0.001]. SUV was significantly correlated with MG score (rho = 0.289, p = 0.017) and marginally with antibodies anti-acetylcholine receptors (rho = 0.129, p = 0.05). At Kaplan Meier analysis, ectopic thymic tissue (p = 0.045) and ectopic germinal centers (p = 0.036) were significant predictors of complete stable remission, but preoperative dichotomized thymic (3.5 or more Vs less) (p = 0.083) and perithymic (2.1 or more Vs less) (p = 0.052) SUVs did not.

**Conclusions:**

Thymic and perithymic SUVs were significantly higher in patients with MG than non-MG and non-neoplastic patients. Thymic SUV was significantly correlated with the presence of germinal centers. Perithymic SUV resulted significantly correlated with the discovery of ectopic active thymic tissue. Neither thymic nor perithymic high SUVs predicted remission.

**Electronic supplementary material:**

The online version of this article (doi:10.1186/s13019-014-0146-0) contains supplementary material, which is available to authorized users.

## Background

Extended thymectomy is considered one of the key points for achieving complete stable remission of myasthenia gravis (MG) [1]-[4]. The persistence of ectopic thymic tissue hosting germinal centers and producing antibodies against acetylcholine receptors (anti-AchR Ab) is supposed to be one of the main reasons of poor outcome after thymectomy [5]-[12]. Positron Emission Tomography (PET) is a radiological device that utilizes 18fluoro-deoxy-glucose (18FDG) to study the metabolism of organs and tissues [13]. It is successfully used to investigate neoplastic masses and for staging purposes by quantifying the pathologic elevation of metabolism in a rapid growing tissue [14].

We hypothesized that in MG patients, the germinal centers contained in both the native and ectopic thymic tissue appear metabolically more active than surrounding tissues. This feature might imply an increased consumption of glucose in these areas and a consequent high standardized uptake value (SUV) on PET.

Herein we analyzed the correlations of SUV with the variables related to the disease and, namely, with the presence of germinal centers in both the native and ectopic thymic tissue. Furthermore, we also investigated the possible influence of SUV on clinical outcome after thymectomy.

## Methods

PET is an investigation based on the intravenous administration of short-duration radionuclides. It is mainly used for diagnostic purposes in neoplastic diseases. For its off-label utilization in a benign condition we asked and obtained a legal permission issued by the “Comitato Etico” (ethical board of our Institution) (prot. No. CT/2004/0396). Each patient was adequately informed about the purposes of the study as well as pros and cons of a radionuclide-based analysis and released written and fully informed consent to the use of PET.

### Patients

Our study included a total of 68 consecutive myasthenic non-thymomatous patients, 37 females and 31 males, aged from 15 to 74 years (mean 41.1 ± 16.6), who underwent extended thymectomy in our multidisciplinary Unit between 2005 and 2012. Major demographic data are summarized in Table 1.

**Table 1:** **Main demographic and clinical features of the study group**

Variables	p-value (n = 68)	
Age, years (median IQR)	39 (25–59)	
Gender		
Male, n. pts (%)	31 (45)	0. 6
Female, n. pts (%)	37 (55)	
Anti AchR Ab, nmol/L (median IQR)	3.1 (2.0-4.0)	
Score MG (median IQR)	23 (14–29)	
Symptom duration		
&<12 months, n. pts (%)	20 (29)	0.69
&12 months, n. pts (%)	48 (71)	
Oropharyngeal symptoms		
&Yes, n. pts (%)	36 (53)	0.91
&No, n. pts (%)	32 (47)	
MGFA classification		
&Class I, n. pts (%)	6 (9)	0.06
&Class II, n. pts (%)	40 (59)	
&Class III, n. pts (%)	22 (32)	
Histology		
&Hyperplasia, n. pts (%)	27 (40)	0.89
&Atrophia, n. pts (%)	23 (34)	
&Normal, n. pts (%)	18 (26)	
Thymic germinal centers, n. pts (%)	33 (48)	
&Ectopic tissue, n. pts (%)	37 (54)	
&Ectopic germinal centers, n. pts (%)	23 (34)	

Values are represented as number of patients (percentage) or median and interquartile range (IQR).

### Study design

This study was designed as a retrospective non-randomized investigation. Only patients with non-thymomatous MG up to Class III according to the MG Foundation of America were included [15]. All patients with thymoma were excluded from the study.

Data were prospectively collected evaluating the surgical details, postoperative complications, histological type, characteristics of postoperative treatment, all information concerning MG (class, MG score, presence of bulbar symptoms, myasthenic crisis, steroid use, blood levels of anti-AchR Ab) and all information regarding the evolution and the date of the possible complete stable remission (CSR). The study entailed the use of archival material such as medical records, radiographs, histological specimens and lab tests, the use of whom was approved by the “Comitato etico” (prot.no. PTV/2011/01522) involved in this investigation.

### MG assessment

The diagnosis of MG was based on clinical features and one or more of the following criteria: positive single-fiber repeated stimulation electromyography, demonstration of circulating anti-AchR Ab and response to edrophonium chloride. A quantitative MG score from 0 (no impairment) to 39 (maximum impairment) was also evaluated pre- and postoperatively [16]. Before thymectomy, all patients were receiving anticholinesterase drugs alone or in combination with steroids (n = 12).

A medical panel composed of neurologists, thoracic surgeons and anesthesiologists discussed the decision and timing for thymectomy. Each patient was clinically stable before surgery. The surgical approach (sternotomy Vs video-thoracoscopy) was chosen by the patients after fully information. In case of increasing weakness or bulbar symptoms, plasmapheresis (n = 2) or intravenous immunoglobulins (n = 3) were administrated immediately prior to surgery.

### PET imaging

The PET/Computed Tomography (CT) system Discovery ST16 (GE Medical Systems, Milwaukee, WI, USA) was used to assess 18FDG distribution in all patients by 3D-mode standard technique in a 128x128 matrix. Filtered back projection was used for image reconstruction. Axial full width at half maximum 1 cm radius 5.2 mm in 3D mode, axial field of view 157 mm. All patients fasted for at least 5 hours before 18FDG intravenous injection. All the subjects were injected with 2.5 MBq/Kg ±10% (210–410 MBq) of 18FDG intravenous and hydrated with 500 mL of intravenous normal saline solution. Whole body PET/CT scan was performed around 60 minutes after 18FDG injection. A low-amperage whole body CT scan for attenuation correction (40 mA; 120 Kv) was performed before PET image acquisition according to standard guidelines [17].

**Figure 1:**
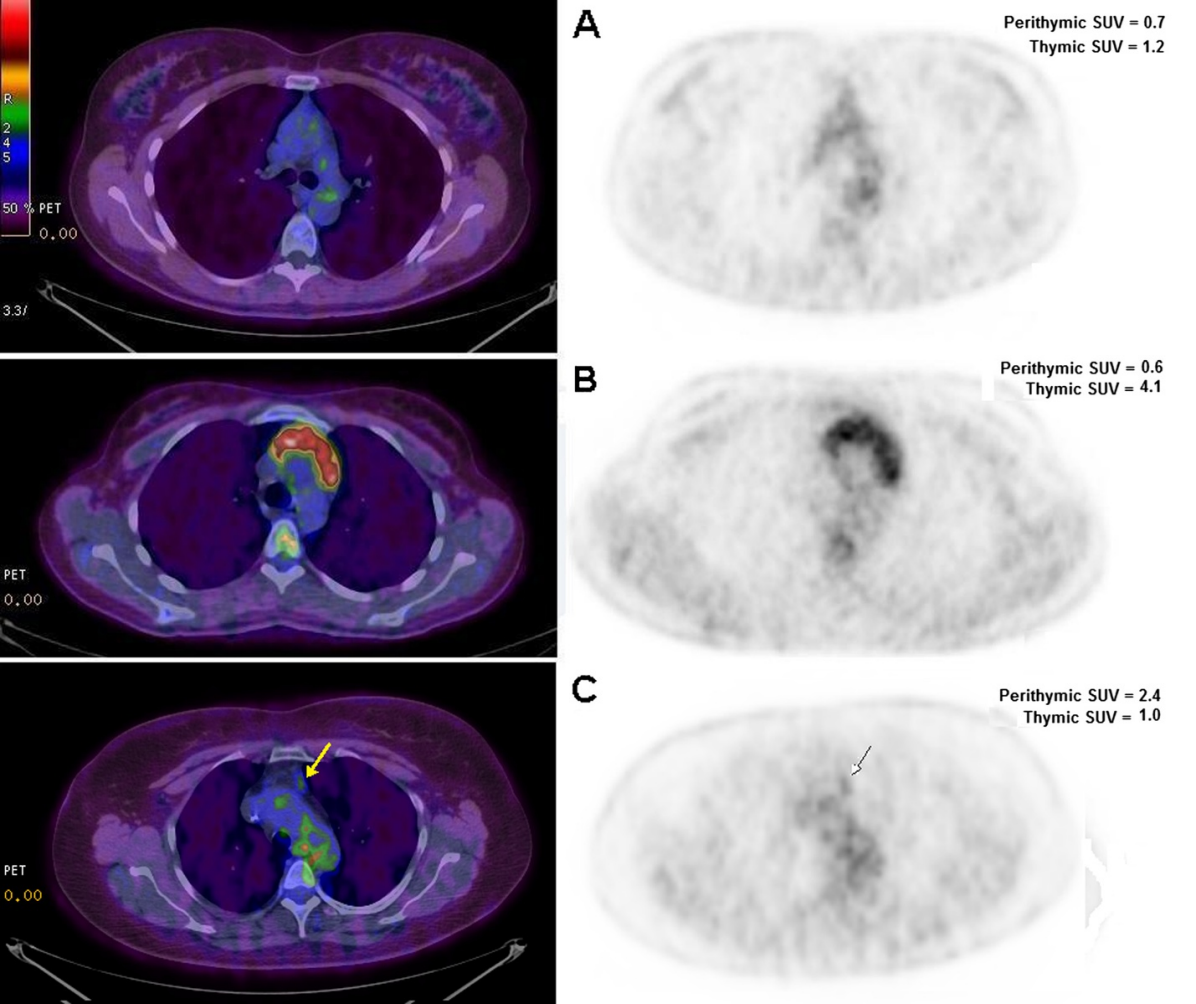
**Positron Emission Tomography images. (A)** normal thymus, **(B)** non-thymomatous myasthenic thymus with elevated standard uptake value, **(C)** non-thymomatous myasthenic thymus with an area of higher standard uptake value in a perithymic region (arrows).

All PETs were reviewed independently by three nuclear med physicians (including OS) Maximum SUV of the thymic and perithymic regions was separately calculated on a dedicated workstation for all the PET/CT examinations. The readers’ independent scores from the PET/CT reading were averaged for each region and the final maximum value was simply indicated in the text as SUV (Figure 1). The images were evaluated without knowledge of patient’s identity, clinical history or subsequent follow-up. For thymus, a volume of interest has been traced on the thymic tissue and all the volumes of interest were furthermore checked in a three planar view in order to exclude the inclusion of unwanted tissues in the area of interest (i.e. vessels, heart wall). For mediastinal perithymic fatty tissue, a standard volume of interest 1x1x1cm (for a total volume of 1 cm^3^) was placed on the mediastinal fat. In order to exclude any partial volume effect, the contrast enhanced CT images were used for a better overall anatomical definition and for a correct volume of interest placement.

### Surgical technique

All the procedures were accomplished under general anesthesia with single-lung ventilation, and were carried out by a single surgeon (TCM). Thymectomy was performed through transsternal (n = 47) or left video-assisted thoracoscopic (n = 21) approaches. The type of procedure was chosen according to patient’s preferences after fully informed consent. Only short-acting, nondepolarizing neuromuscular relaxants were occasionally used. Pyridostigmine was administered during the operation and up to 12 hours afterwards through a nasogastric tube. All surgical steps are described elsewhere [10],[18]. Resection routinely included perithymic fat tissue from anterior mediastinum, neck, aorto-caval groove, aorto-pulmonary window, retro-innominate space, and both cardiophrenic angles. Thymectomy was considered extended according to Sonnet and Jaretzki [19].

At the end of the operation, we encouraged early extubation of the patients and rapid physiotherapy. Pain control was preferentially performed by means of oral analgesics only. Anticholinesterase drugs and steroids were administrated according to clinical status.

### Thymic specimen evaluation

Histological examination of all excised tissue was described elsewhere [10]. Classic hematoxylin and eosin-stained sections were firstly examined under low magnification (×20). Ectopic thymus was randomly researched by taking 1 from every 3 sections of fat perithymic tissue inclusion. The presence of germinal centers, also named active thymic tissue, was investigated with monoclonal antibodies against CD23 [10]. Mouse monoclonal anti-human antibodies specific for CD23 (clone 1B12, Novocastra, UK) were used at a 1:100 dilution. Cytoplasmic staining with anti-human CD23 was scored as positive by an experienced pathologist.

### Follow-up

Patients were followed in the dedicated MG unit by the same team of surgeons and neurologists every 3 months within the first year and then every 6 months. Patients continued to receive medical therapy (i.e., anticholinesterase or steroids), which was progressively reduced and eventually quitted in the absence of symptoms. No specific immunosuppressive drug was used. The therapeutic effect and symptomatic response to the different therapies was established for each patient according to the MG Foundation of America (MGFA) post-interventus status classification [15]. CSR was defined as no symptoms or signs of MG at careful examination for at least 12 months achieved without therapy. Improvement was defined as amelioration of MG-related symptoms (clinical) or a long-lasting decrease in MG therapy (pharmacologic). Patients whose MG symptoms or administered medication dosages were stable were scored as ‘unchanged’. Equally, those patients whose symptoms became more serious or required an increase in medical therapy, were all defined as having ‘worsened disease’. PET was routinely repeated at one year follow-up and evaluated by the same physicians.

### Statistical analysis

Preliminary descriptive analysis of variance was performed for all variables to identify non-normal distribution or incorrect data. Non-parametric tests were used and data were expressed as median and interquartile range. All analyses were performed in SPSS statistical package (SPSS Inc., Chicago, IL). Results were considered significant for p < 0.05. Continuous variables were correlated by Spearman test. Prognostic evaluation was conducted by univariate and multivariate analysis selecting as primary outcome the improvement at the end of follow up.

CSR rate was defined as number of patients asymptomatic and off-medications for at least 12 months over total number of patients evaluated. Kaplan-Meier estimate of time to CSR was determined. Time to CSR was defined as time from operation day to first date of CSR. Similarly, those patients who had not achieved CSR were censored and their consoring time was defined as time from operation to most recent patient contact. Univariate and multivariate survival analyses were performed by the log rank test and Cox regression, respectively, in order to evaluate the impact of several preoperative and operative factors on time to CSR. To determine the best cutoff value for the thymic and perithymic SUV, we performed the Kaplan-Meier analysis for CSR using several different cutoff values, choosing the one returning the larger separation. The choice was confirmed by the analysis of martingale residuals from the null Cox model (not showed).

## Results

### Perioperative complications

No patient died in the perioperative period. Two patients (4.2%) suffered from pneumonia with pleural effusion and one patient who had an episode of atrial fibrillation with spontaneous resolution in the second post-operative day. Mean hospital time was 4.5 ± 2.1 and 2.6 ± 2.2 days, respectively (p = 0.03). One patient (2.1%) developed a postoperative myasthenic crisis, which needed intubation and subsequent treatment with plasmapheresis.

### Histological findings

Histological examination showed the presence of atrophic thymus gland in 23 patients (34%), hyperplastic in 27 patients (40%) and normal thymic tissue in 18 patients (26%). The presence of thymic germinal centers was detected in 43 (63%) patients. These centers were significantly associated with hyperplastic thymus [21/27 (77%) Vs normal 11/18 (61%) Vs atrophic 1/22 (4%), p < 0.0001]. Ectopic thymic tissue was detected in 37 patients (54%) and most of these (23/37, 62%) presented ectopic germinal centers. They were significantly associated with atrophic thymus [14/23 (61%) Vs hyperplastic 4/27 (15%) Vs normal 5/18 (28%), p = 0.002]. Neither circulating anti-AchR Ab nor MG score were significantly correlated with thymic histology (data not showed).

### SUV assessment

**Figure 2:**
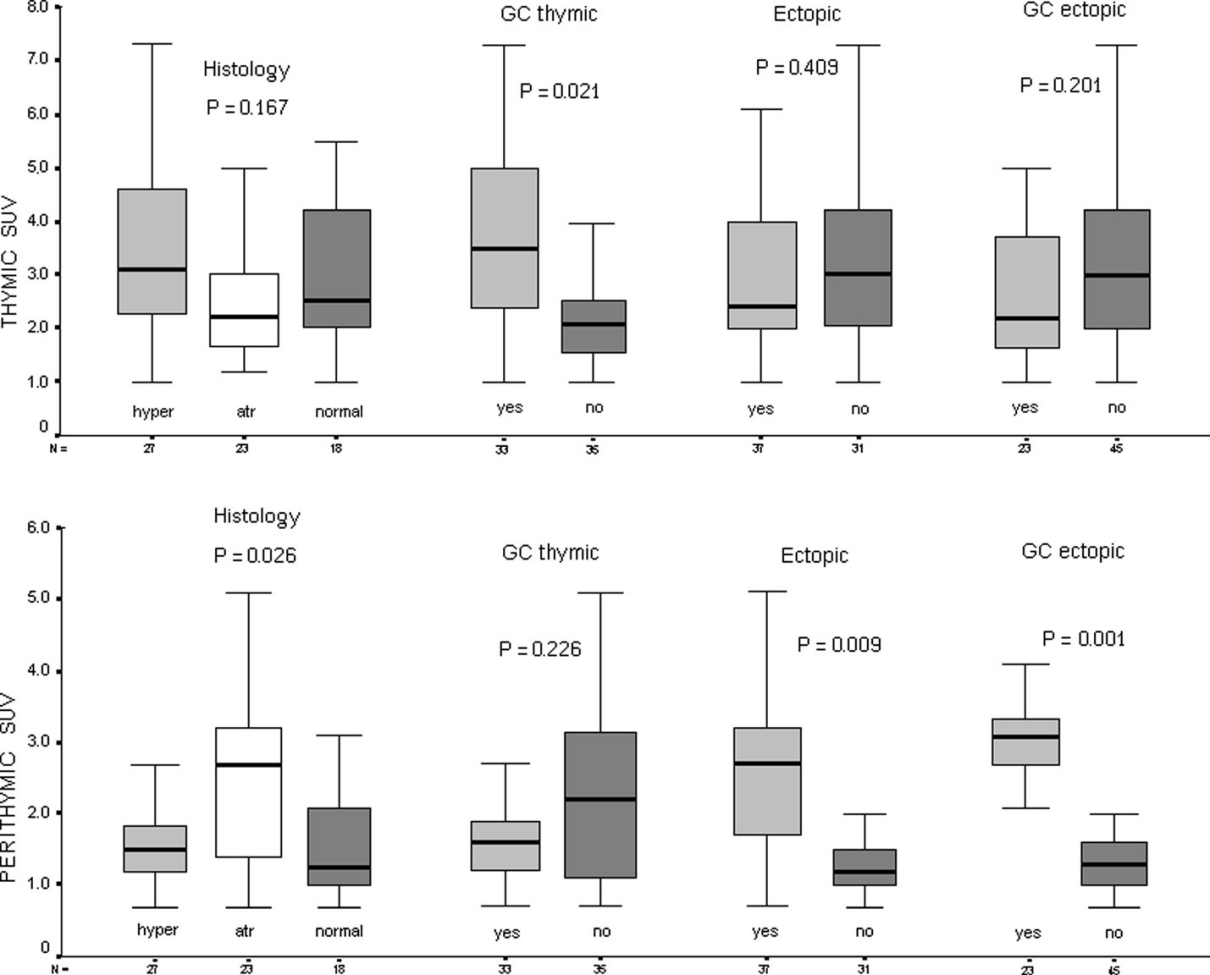
**Thymic and perithymic SUV correlations with major pathologic variables.** Data are indicated as median and interquartile ranges. Significance was calculated by Kruskal-Wallis or Mann–Whitney when indicated.

MG patients showed an increased SUV in both thymic and perithymic tissue compared to results obtained in 108 same-aged non-myasthenic and non-oncologic patients who performed PET in the same period: thymic SUV 2.5 (2.2-4.1) Vs 1.2 (0.7-1.6), p = 0.009 and perithymic SUV 1.6 (1.1-2.7) Vs 0.9 (0.6-1.4), p = 0.02. Thymic SUV was significantly higher in presence of thymic germinal centers [3.5 (2.4-5.0) Vs 2.1 (1.4-2.5); p = 0.021] (Figure 2). Furthermore, it resulted significantly correlated with MG score (rho 0.289; p = 0.017), while it was only marginally correlated with titer for anti-AchR Ab (rho 0.129; p = 0.05). No additional associations between thymic SUV and other clinical variables was detectable.

Perithymic SUV associated to atrophic thymus was significantly higher (p = 0.021) [2.7 (1.4-3.2)] compared to hyperplastic [1.6 (1.2-3.3)] or normal [1.3 (0.9-2.0)] thymus (Figure 2). In addition, it was even further increased in presence of ectopic thymic tissue [2.7 (1.7-3.2) Vs 1.3 (1.0-1.5); p = 0.009], especially when housing germinal centers [3.1 (2.7-3.5) Vs 1.3 (0.9-1.7); p = 0.001] (Figure 2). Increased perithymic SUV allowed us to remove ectopic thymic islets sited in aortopulmonary window (n = 2), anterior mediastinum (n = 1) and right pericardiophrenic angle (n = 1) in patients with atrophic thymus. All these islets were unsuspected on the basis of a simple CT scan.

At one year follow-up, PET showed a significant decrement of SUV activity in residual mediastinal area [1.1 (0.8-1.8); p = 0.042] compared to preoperative perithymic activity.

### Long-term outcomes

**Figure 3:**
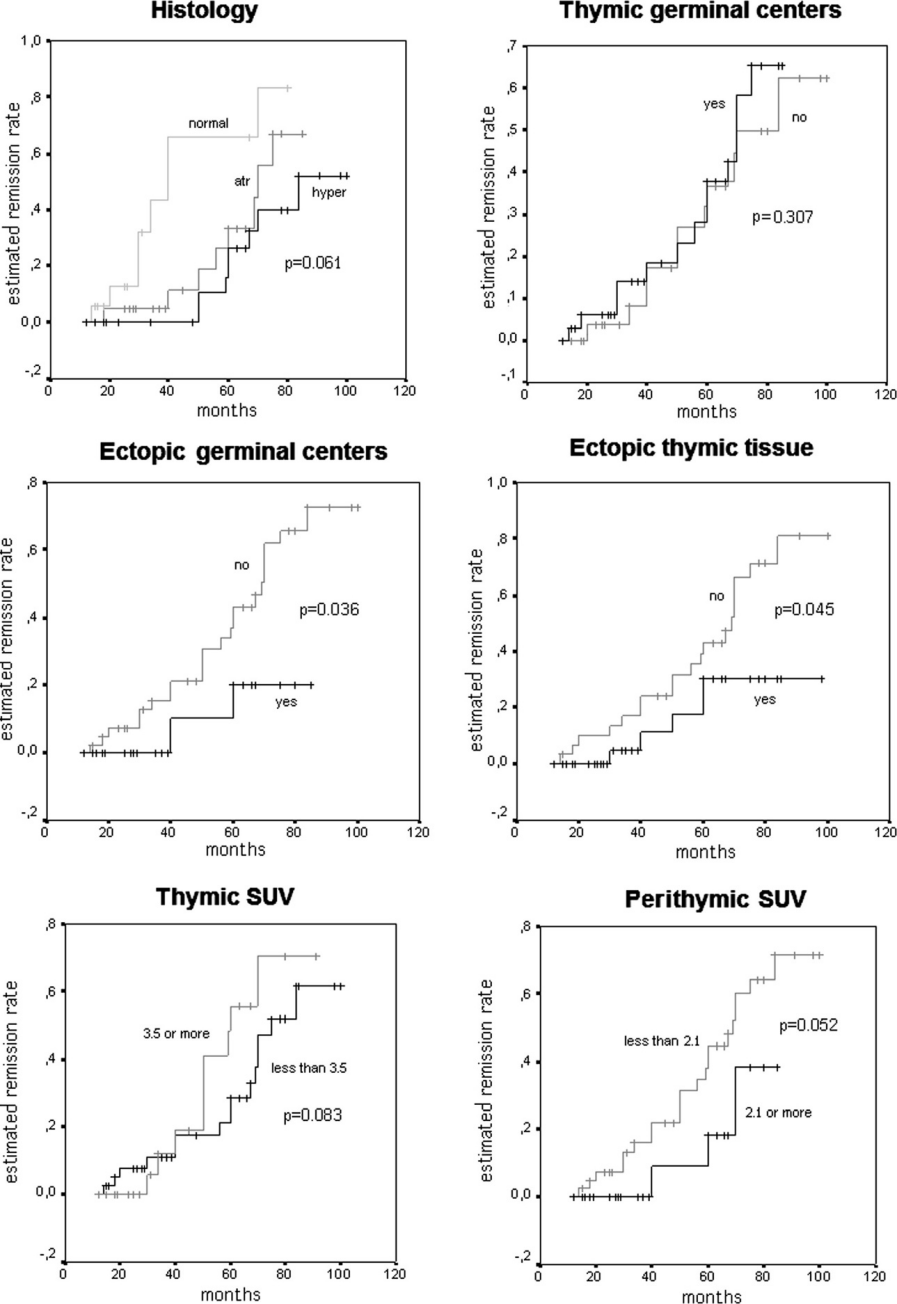
**Kaplan-Meier remission curve and log rank test for different variables.**

No patient was lost during follow-up. The average time for each patient was equal to 49 (25–68) months (range 12–96). During the study, 42 patients (62%) demonstrated an improvement of the MG symptoms according to the MGFA post-interventus status. CSR occurred in a total of 24 subjects (35%), 17 of whom within 60 months from the procedure (Table 2). The Kaplan-Meier curves are shown in Figure 3. CSR rate was 31% at 60 months and 64% at 90 months from thymectomy, respectively. Median time to CSR was 73 months. Histology (p = 0.061), MGFA Class (p = 0.187) and surgical approach (p = 0.674) did not influence CSR. Whereas the presence of ectopic thymus tissue (p = 0.045) especially when hosting germinal centers (p = 0.036) were significantly correlated with CSR.

**Table 2:** **Univariate analysis of factors predicting the improvement or complete remission within 5 years**

	MGFA Postinterventus status improvement			Complete Remission		
Variables	Yes (n = 42)	No (n = 26)	p-value	Yes (n = 17)	No (n = 51)	p-value
Gender						
&Male	18	13		8	23	
&Female	24	13	0.071	9	28	0.888
Symptom duration						
&<12 months	12	8		10	10	
&12 months	30	18	0.068	7	41	0.054
Oropharyngeal symptoms						
&Yes	22	14		8	28	
&No	20	12	0.424	9	23	0.342
MGFA class						
&Class I	3	3		1	5	
&Class II	24	16	0.674	10	30	0.868
&Class III	15	7		6	16	
Surgical approach						
&Sternotomy	29	18		11	36	
&VAT	13	8	0.123	6	15	0.449
Histology						
&Hyperplasia	18	9		5	22	
&Atrophia	12	11	0.045	5	18	0.274
&Normal	12	6		7	11	
Thymic germinal centers						
&Yes	23	10		8	25	
&No	19	16	0.191	9	26	0.889
Ectopic thymic tissue						
&Yes	18	19		5	32	
&No	24	7	0.024	12	19	0.024
Ectopic germinal centers						
&Yes	9	14		2	21	
&No	33	12	0.009	15	30	0.037

Preoperative thymic SUV was not clearly associated with CSR (effect shown in Figure 3 using a cutpoint at 3.5, p = 0.083). Higher values of perithymic SUV (2.1 or more Vs less than 2.1) were associated with lower incidence of CSR although this effect did not reach conventional statistical significance (p = 0.052).

Cox regression analysis showed that ectopic tissue with germinal centers was the most powerful yet not-significant prognostic factor (p = 0.065, Wald = 3.446, Exp = 3.952, 95% CI = 0.926-1.671).

## Discussion

PET has been already used to study the thymus in the radiologic evidence of an abnormal mass [14]. However, PET has never used for the assessment of the immunological activity in a non-thymomatous thymus in patients with MG. According to our hypothesis, these patients may present a metabolically active mediastinal area that produces anti-AchR Ab and these regions might reveal a more elevated glucose uptake on PET.

Our data evidenced higher SUVs in both thymic and perithymic areas from MG patients compared to normal standard values of our control group. Furthermore, we highlighted a correlation between the thymic SUV and the presence of thymic germinal centers. Thymic SUV also disclosed a correlation with MG score and marginally with anti-AchR Ab titer.

We experienced a significant correlation between perithymic SUV and the presence of ectopic thymic tissue, showing higher values whenever this tissue hosted germinal centers. Furthermore, in atrophic histology perithymic values were higher than the actual thymic ones. The discovery of ectopic thymic tissue [9] and mainly of ectopic germinal centers [10],[11],[20] in surgical specimens may obstacle CSR.

This potential ability of PET/CT in discovering tissue with intense immunologic activity might delineate a preoperative role for this investigation. Patients disclosing intense thymic activity might be operated on with good results. Conversely, the evidence of consistent perithymic activity should alert the surgeon about the need of a more extended resection volume and the possibility of more difficult remission.

PET has still a major limitation in the resolving power of the method [21] and we mainly attribute to this inconvenience the lack of significance in predicting CSR. SUV measure of perithymic areas may be somewhat undefined due to the indetermination of perithymic boundaries and to heterogeneity of anatomical structure in it. Furthermore the current devices can only highlight areas in which the concentration of more germinal centers exceeds the extension of the middle square centimeter [22]. A germinal center is a very small set of cells (i.e. thymocytes and lymphocytes) that by itself constitutes a microenvironment with its metabolism and its source of supply nutritive. This could explain why in the hyperplasia, where the germinal centers are organized into areas, the SUV is higher, while in the perithymic fat, even in presence of ectopic germinal centers, the SUV is less high. This imprecise evaluation might justify why an elevated SUV was not correlated to a significant statistical difference in symptoms amelioration and CSR.

The retrospective nature is an evident limitation of this study, however this should be considered only as an observational study to assess the feasibility of future more structured investigations. Surgical group allotment based on the patient’s choice is usually considered a major bias but, as above mentioned, the comparison of two different approaches was not the purpose of the study. We have already mentioned the limitation of image resolution and the difficulty in outlining and measuring SUV in perithymic areas. These potential flaws were mitigated by the homogeneous evaluation conducted by a task force of experienced PET evaluators. The increasing PET ability to trace metabolic maps might become in a relatively short future a helpful device in detecting the presence of these active areas and better orientating surgical resection.

## Conclusions

We can conclude that SUV resulted more elevated in MG patients in both thymic and perithymic areas. Thymic SUV was significantly correlated with the presence of germinal centers. Perithymic SUV resulted significantly correlated with the discovery of ectopic active thymic tissue. Unfortunately the resolution-power of PET did not yet allow the demonstration of a correlation with MG outcomes. On these bases and due to the high cost of the exam at present there is not a consistent reason to use it out from study protocols. Nevertheless the potential role of SUV in evidencing active ectopic tissue might be worthy of further investigations.

## Authors’ original submitted files for images

Below are the links to the authors’ original submitted files for images. Authors’ original file for figure 1 Authors’ original file for figure 2 Authors’ original file for figure 3

## Abbreviations

MG: Myasthenia gravis
SUV: Standardized uptake value
PET: Positron emission tomography
anti-AchR Ab: Antibodies against acetylcholine receptors
18FDG: 18fluoro-deoxy-glucose
CSR: Complete stable remission
CT: Computed tomography
MGFA: Myasthenia Gravis Foundation of America
