# Supplementary figures and images for: May positron emission tomography reveal ectopic or active thymus in preoperative evaluation of non-thymomatous myasthenia gravis?
Source: J Cardiothorac Surg. 2014 Sep 5;9:146. doi: 10.1186/s13019-014-0146-0 (PMC4174284; doi:10.1186/s13019-014-0146-0)

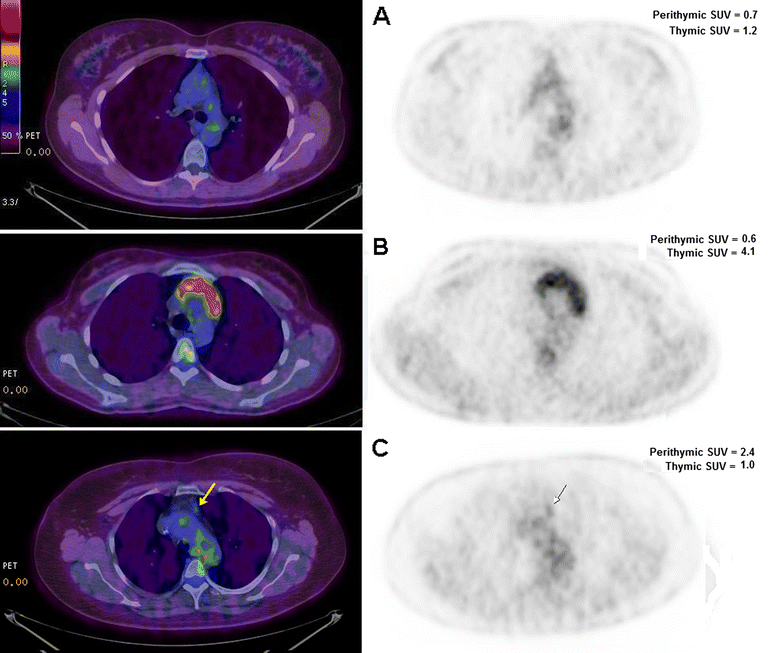

Supplement: Supplementary file 1 — Authors’ original file for figure 1 [file 13019_2014_146_MOESM1_ESM.gif]

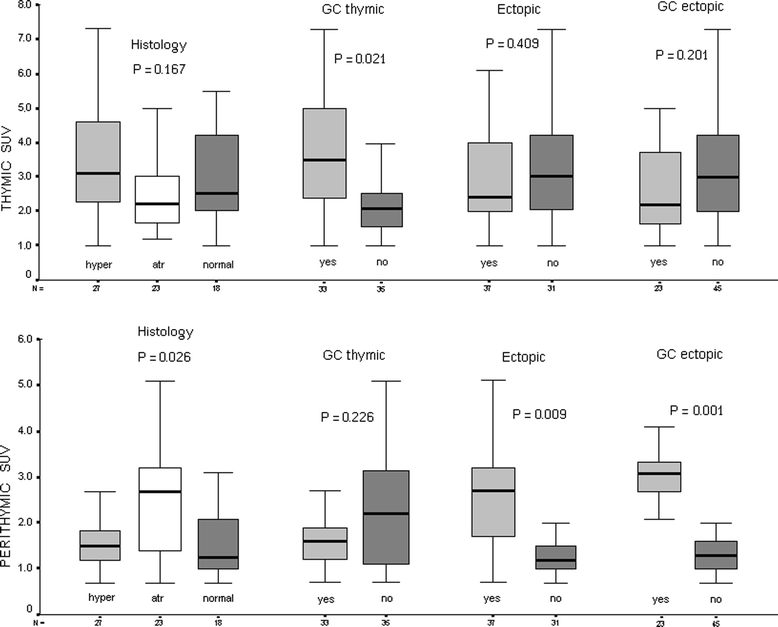

Supplement: Supplementary file 2 — Authors’ original file for figure 2 [file 13019_2014_146_MOESM2_ESM.gif]

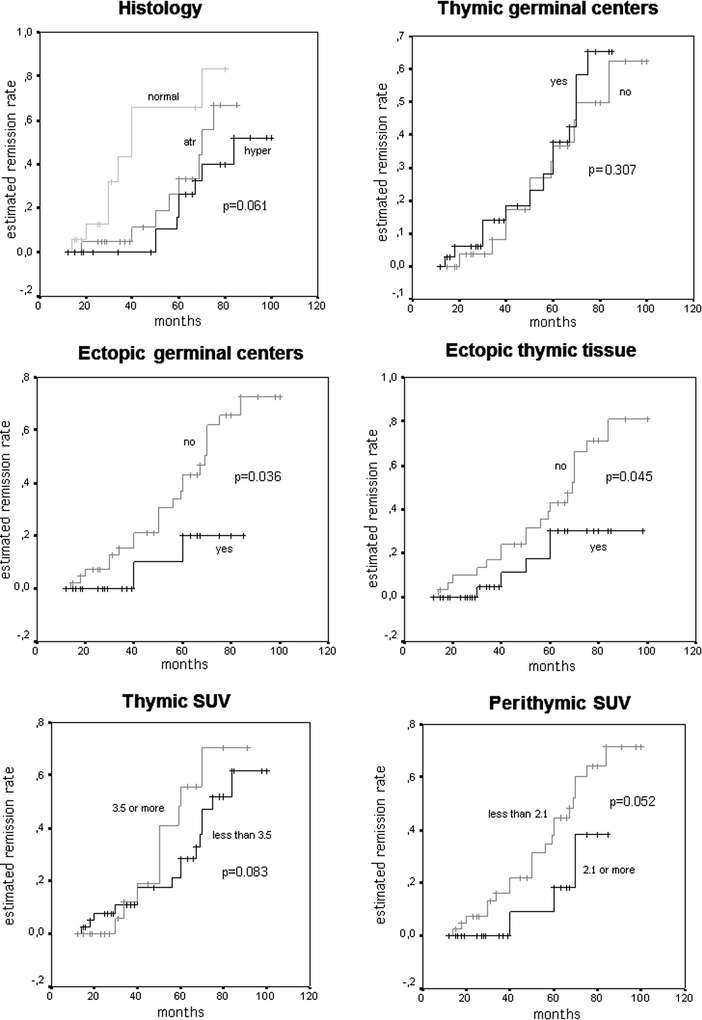

Supplement: Supplementary file 3 — Authors’ original file for figure 3 [file 13019_2014_146_MOESM3_ESM.gif]
